# Isolation, Identification and Characterization of an Electrogenic Microalgae Strain

**DOI:** 10.1371/journal.pone.0073442

**Published:** 2013-09-03

**Authors:** Yicheng Wu, Kai Guan, Zejie Wang, Bing Xu, Feng Zhao

**Affiliations:** Institute of Urban Environment, Chinese Academy of Sciences, Xiamen, People’s Republic of China; RMIT University, Australia

## Abstract

Extracellular electron transfer involving microbes is important as it closely reflects the ability of cells to communicate with the environment. However, there are few reports on electron transfer mechanisms of pure microalgae and a lack of any model alga to study the transfer processes. In the present study, nine green microalgae species were isolated from wastewater and characterized in terms of their ability to transfer electrons between cells and an electrode. One species showed direct electron transfer via membrane-associated proteins and indirect electron transfer via secreted oxygen. The microalga was identified as *Desmodesmus* sp. based on phylogenetic analysis and electron microscopy. Electrochemical tests demonstrated that *Desmodesmus* sp. was able to act as a cathodic microorganism. Stable current densities of −0.24, 35.54 and 170 mA m^−2^ were achieved at potentials of +0.2, −0.2 and −0.4 V, respectively, under illumination. Dissolved oxygen concentration measurement showed gradients within the microalgae biofilm: 18.3 mg L^−1^ in light decreasing to 4.29 mg L^−1^ in the dark. This study diversified the exoelectrogen library and provided a potential model microalga to explore the associated mechanism of extracellular electron transfer.

## Introduction

Bioelectrochemical systems (BES) are paid increasing attentions because of their ability to provide power and to treat wastewater with the assistance of electroactive microorganisms [1]. Recently, the function of BES has been expanded to generate value-added products [2]. Microalgae, as one of the most abundant microorganisms, are able to access solar energy to split water, providing electrons and oxygen [3], [4]. Adoption of microalgae in BES can produce organic matter and simultaneously consume carbon dioxide on an electrode surface [5], [6]. Other functions, such as N, P absorption [7], biodiesel production [8] and biomass supply [9] increase the potential application of microalgae in renewable energy generation and wastewater treatment.

Understanding of extracellular electron transfer will be helpful in optimizing practical applications and developing new functions for BES. The electron transfer mechanism for bacteria has been proposed through analysis of model organisms, e.g. *Geobacter* and *Shewanella* genera [10], [11]. Direct electron transfer is via electroactive proteins, while indirect transfer is with the aid of redox mediators secreted by bacteria. However, electron transfer between electrodes and microalgae has not been addressed [12], which limits the available information on optimization and extension of the functions of electrode-microalgae interactions.

In view of increasing interest in adopting photosynthetic microalgae, a more comprehensive insight into the electron transfer mechanism between microalgae and electrode is of interest. Moreover, a model for pure algae is required to study the mechanism. In the present study, the novel microalgae strain Desmodesmus sp. isolated from wastewater was investigated in terms of electron transfer mechanism and application to enhance the current generation under various conditions.

## Materials and Methods

### Isolation of Axenic Uniclonal Cultures

Wastewater sampled from the Jimei wastewater plant (Xiamen, China) was serially diluted from 10 to 10^5^ times and plated on sterile agar plates prepared by adding 1.5% agar to BG11 medium [13]. In this study no specific permissions were required for the location, which is one of the public wastewater treatment plants in Xiamen. Field studies were related to nine green microalgae species which were isolated from the wastewater; no endangered or protected species were involved in this study. Strains were made axenic by continual sub-culturing in BG11 agar plates supplemented with ampicillin and kanamycin. In the absence of fungal or bacterial contamination, individual colonies were cultured in liquid BG11 media for further analysis. The alga strain was grown in BG11 medium at 28±1°C and under a light-dark regime of 12∶12 h. Cold fluorescent lamps were used for illumination.

### Electrochemical Analysis

Cyclic voltammetry (CV) was performed using a three-electrode system with glassy carbon electrode (3 mm diameter) as working electrode, a platinum wire as counter electrode, and Ag/AgCl (3 M KCl) as reference electrode. To discover the electron transfer mechanism, the working electrode was covered with isolated microalgae using Nafion ionomer (5% dispersion) as a binder. CV traces were determined in the potential range of −0.8 to +0.8 V at a scan rate of 10 mV s^−1^ (Autolab 302N potentiostat, Netherlands). Phosphate buffer (0.05 M, pH 7.2) was deoxygenated by purging with nitrogen gas for 30 min before, and during the measurement.

For chronoamperometric tests, the BES were prepared of a 100 mL glass bottle with Ag/AgCl (3 M KCl) as reference electrode and graphite felt (Haoshi, China) as both working and counter electrodes, with a projected surface areas of 4 cm^2^ (2×2 cm) and 6 cm^2^ (1.5×4 cm) respectively. BG11 medium was introduced to the BES to inoculate the microalga strain. To evaluate current generation, potentials of −0.4, −0.2 and +0.2 V vs. Ag/AgCl were maintained with an electrochemical workstation (CHI1000B, CH Instruments Inc., China). The effect of illumination on current generation was further examined by holding the working electrode at −0.4 V under illumination (1500 lux). The data points were recorded every 5 min. When operated in dark, the systems were wrapped with aluminum foil. Control experiments (i.e. no addition of inoculum) were simultaneously performed. Titanium wires were employed for connections and terminal. The experiments were conducted at 28°C in an illumination incubator with a cool white fluorescent light source. All the electrochemical experiments were conducted in triplicate.

### Electron Microscopic Analysis

SEM: The strain A8 cells were fixed in 2.5% glutaraldehyde at 4°C for 24 h, rinsed several times in 0.05 M phosphate buffer (pH 7.2), and serially dehydrated in 20%, 35%, 50%, 75%, 90% and 100% ethanol solution for 5 min. After dehydration, the samples were dried using a critical point drying process in liquid CO_2_ (Tousimis Samdri®-PVT-3D, United States), then sputter-coated with Au and examined using an S-4800 Field Emission Scanning Electron Microscope (Hitachi, Japan).

TEM: The strain A8 cells were harvested by centrifugation (4500 rpm, 10 min), washed with 0.05 M phosphate buffer (pH 7.2), fixed in 2% glutaraldehyde for 24 h, and post-fixed with 1% osmium tetroxide for 30 min. The material was then dehydrated in the ethanol series and embedded in low-viscosity resin. Sections were cut with a glass knife on an EM UC7 ultramicrotome (Leica, Germany). Semi-thin sections were stained with blue toluidine. Ultrathin sections were placed on copper grids, and then stained with uranyl acetate and lead citrate. Observations were carried out with an H-7650 transmission electron microscope (Hitachi, Japan).

### DNA Extraction, PCR Amplification and Sequence Analysis

Total genomic DNA was extracted from the isolated strain A8 using a plant genomic DNA extraction kit (Bioteke, China), according to the instructions of manufacturer. Amplification of ITS1, 5.8S and ITS2 regions of the ribosomal DNA was performed by PCR using the following primers: ITS4 (5′-GGAAGTAAAAGTCGTAACAAGG-3′) and ITS7 (5′-TCCTGGTTAGTTTCTTTTCC-3′) [14]. PCR amplification was carried out in Mastercycler gradient PCR apparatus (Eppendorf, Germany) with an initial denaturation of DNA for 5 min at 95°C, followed by 30 cycles of 40 s at 95°C, 40 s at 56°C, and 2 min at 72°C, then final extension for 10 min at 72°C, and 4°C hold. PCR products were purified using a DP 1601 Gel purification kit (Bioteke, China).

The purified products were ligated into pMD®19-T vector (Takara, China), and cloned into chemically competent *E. coli* cells. Plasmids were isolated from randomly selected clone with a MiniBEST plasmid purification kit (Takara, China). Three plasmid inserts were then sequenced in both directions using an ABI 3730 DNA sequencer (Applied Biosystems, USA).

### Phylogenetic Analysis

BLAST (Basic local alignment search tool) was used to compare the rDNA ITS sequences of A8 to sequences in the GenBank database and calculated the statistical significance of the matches, the most closely related 10 species were selected for phylogenetic analysis [15], [16]. The rDNA ITS sequences of A8 and closely related strains were aligned using Clustal X software [17]. The Neighbor-Joining method in the software Mega 4.1 was used to construct the phylogenetic tree [17], [18]. Confidence estimates of the branching order were determined by bootstrap analysis with 1000 replicates.

### Dissolved Oxygen (DO) Determination

The oxygen concentration gradient within the microalgae biofilm was determined using a DO microelectrode (Unisense, Denmark). A stepper motor was used to control the movement of the microelectrode. The signals were acquired by a Microsensor Multimeter (Unisense, Denmark). Each measurement was started 200 µm above the biofilm surface and the step size of the microelectrodes was set as 50 µm controlled by the motor. At each measurement point, there was an initial dwell of 30 sec and then measurement for 5 sec. Three points on the biofilm were randomly examined. The temperature was controlled at 28°C.

## Results and Discussion

### Light Microscopic Observation of the Isolated Microalgae

**Figure 1:**
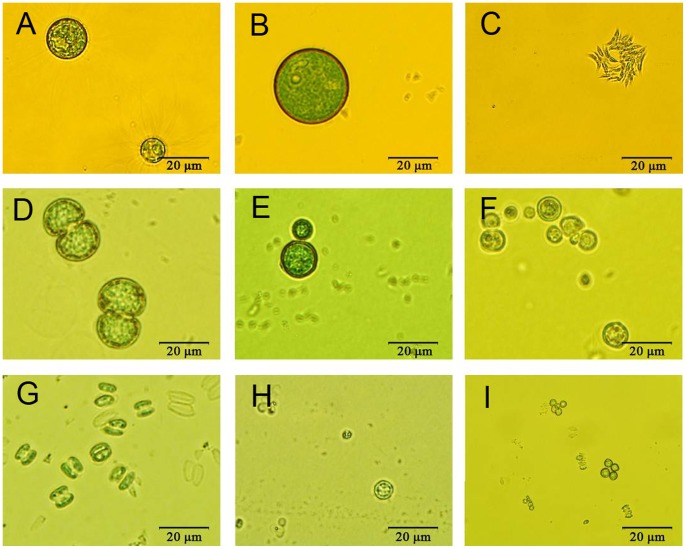
Light microscope (1000×) pictures of microalgae isolates.

Wastewater was sampled onto agar plates. Green microalgae clones were picked up after a few cycles of agar plate spreading. Nine species were isolated. The morphology of isolated strains was observed with light microscopy (Figure 1). Five species (Figure 1b,1 e,1 f, 1h and 1i) were spheres with different size, of which the largest diameter is about 30 µm (Figure 1b) and the smallest one is about 3 µm (Figure 1i). The isolated unicellular alga shown in Figure 1a has a spherical cell body with spiny projections, and a diameter of 18–20 µm, which are the diagnostic characteristics of *Golenkinia*. From Figure 1d, the cells are deeply divided in the middle by a short isthmus, and the two semi-cells are oval. Microscopic analysis of the samples allowed preliminary identification of this isolate as genus *Cosmarium*. Two *Desmodesmus* with different size and form were isolated (Figure 1c and 1g); one is a four-cell coenobia which is crescent-shaped, and the other is two-cell coenobia which is the most frequently with oven form and denoted A8.

### Electron Transfer Mechanism

**Figure 2:**
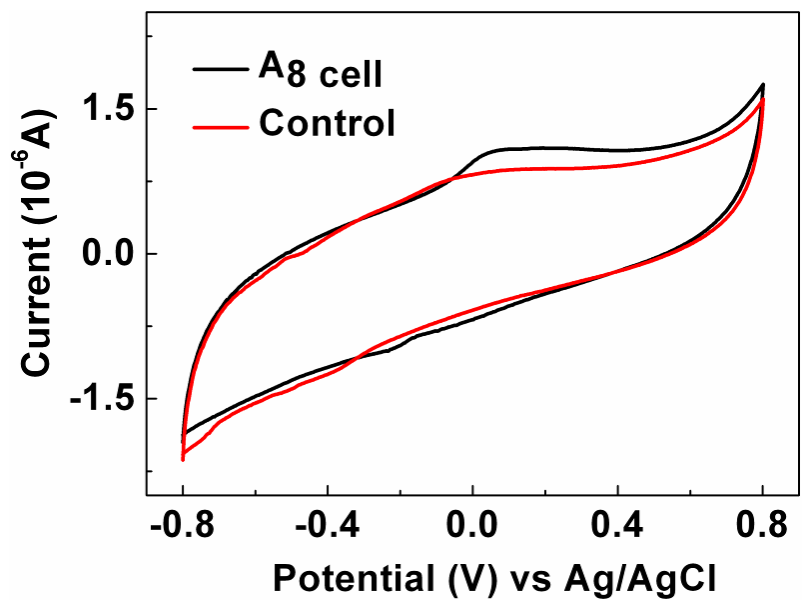
Cyclic voltammograms of strain A8 on glassy carbon under anaerobic conditions.

To evaluate the redox activity of the nine isolates, CV measurements for the isolated microalgae on glassy carbon were carried out under anaerobic conditions. The supernatant of the microalgae culture solutions were also collected at the end of the batch experiment. As shown in Figure 2, an oxidation peak was observed in the potential range of +100 to +200 mV for the isolated alga A8 under nitrogen conditions, but no peaks were observed when the supernatant was tested (Figure S1 in File S1). The ability of electron transfer at electrode/biofilm interfaces is the characteristic to distinguish exoelectrogens. For bacteria (i.e. *Shewanella oneidensis* MR-1 [20] and *Geobacter sulferreducens* [21]), mechanisms including indirect transfer via flavin and direct transfer via proteins were reported [19]; some cytochromes of terminal reductases are involved in electron transfer processes. For microalga A8, since there was no electrochemical response of the supernatant, a proposed mechanism is that some proteins such as cytochromes on the outer membrane may be involved in direct electron transfer involving A8. After electrochemical testing, the A8 strain was adopted for further study.

### Morphological Characterization

**Figure 3:**
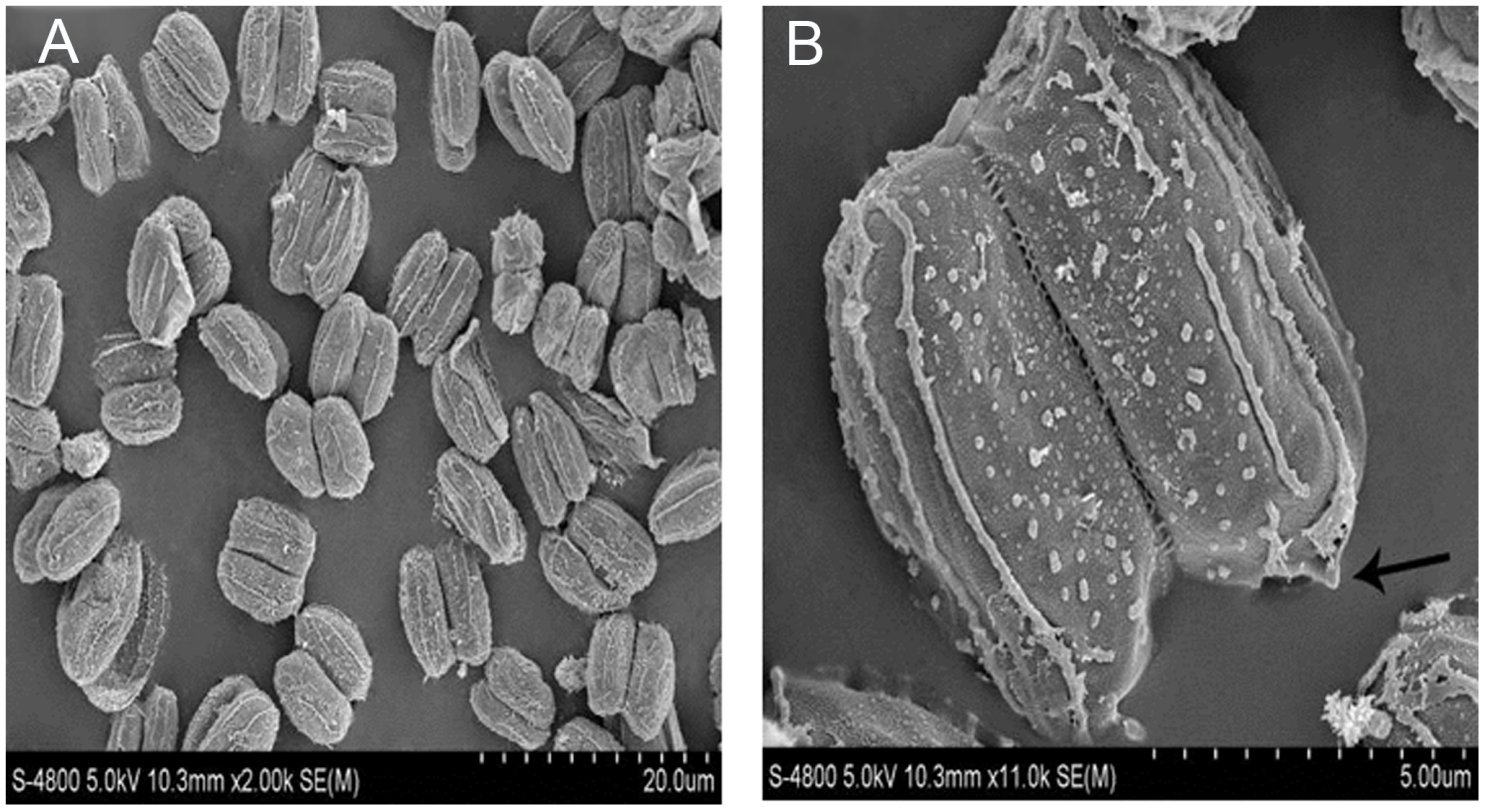
Scanning electron micrographs of A8 cell. A: Magnification×2000; B: Magnification×11000. The strain A8 shows small dents observed at the poles (shown in thin arrow).

For the isolated strain A8, the morphological characteristics included the presence of dents at the pole of the coenobia and ribs on the cell surface (Figure 3a and 3b), which are characteristic to *Desmodesmus* [22], [23]. The most striking feature of the cell wall ornamentation is the uninterrupted pattern of ribs and the absence of large warts, which are also in line with the diagnostic characters of *Desmodesmus* [24]; the presence of spines and the surface morphology of the cell wall are two important diagnostic characteristics to distinguish *Scenedesmus* and *Desmodesmus* species [23]. In cellular suspensions with moderate density, two-cell coenobia were the most frequent, but singular cell coenobia was rarely observed (Figure S2 and S3 in File S1).

**Figure 4:**
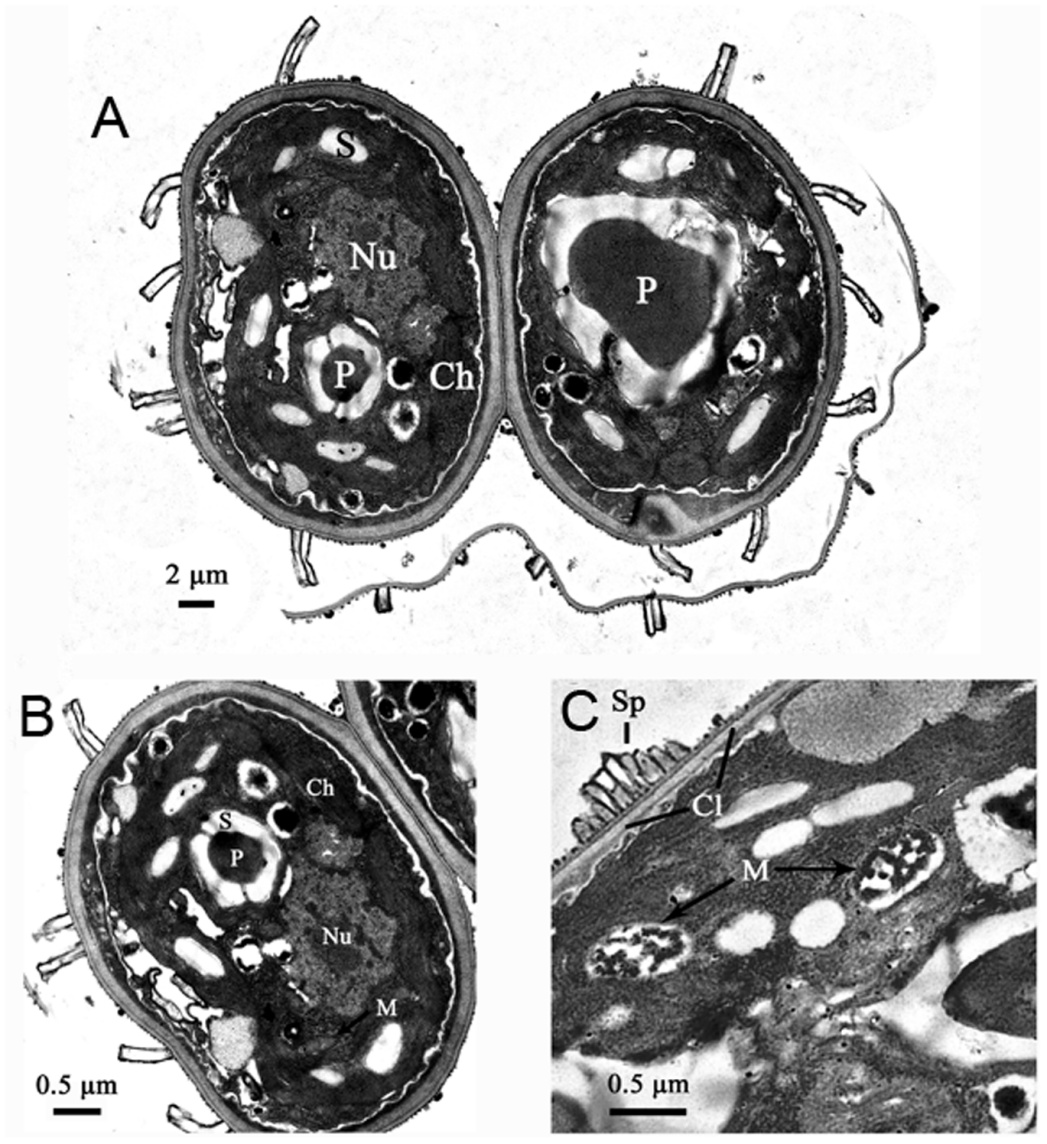
Transmission electron micrographs of A8 cell. A: Transverse section through two-celled coenobia, Bar = 2 µm; B: Transverse section through a cell, Bar = 0.5 µm; C: Part of a cell wall, Bar = 0.5 µm (Abbreviations: Ch = chloroplast; Cl = cellulosic layer; M = mitochondrial profiles; Nu = nucleolus; P = pyrenoid; S = starch; Sp = spine.).

Figure 4a shows the overall image and cross-sections through the cell of A8. The TEM images showed that the A8 is an oval-shaped microalga, ranging from 3 to 5 µm in width and 8 to 12 µm in length (Figure 4b). Abundant chloroplasts are located peripherally, with sparse opaque thylakiod lamellae, and occupy almost half of the cell volume. A well-developed pyrenoid surrounded by a ring of starch granules is observed. Next to the starch grains, a nucleus is located, in which a nucleolus can be seen. Dictyosomes occur in a close proximity to the nucleus. Several mitochondrial profiles with different size are also easily seen in the cytoplasm. The outermost cell wall layer surrounding the entire coenobium is observed, and the thick cell wall consists of at least two layers. The inner, thickest layer is the cellulose layer (Cl) (Figure 4c). The outer layer, named the “warty layer”, has wall ornamentation consisting of rosettes and spines [25].

### Sequences and Phylogenic Analysis

**Figure 5:**
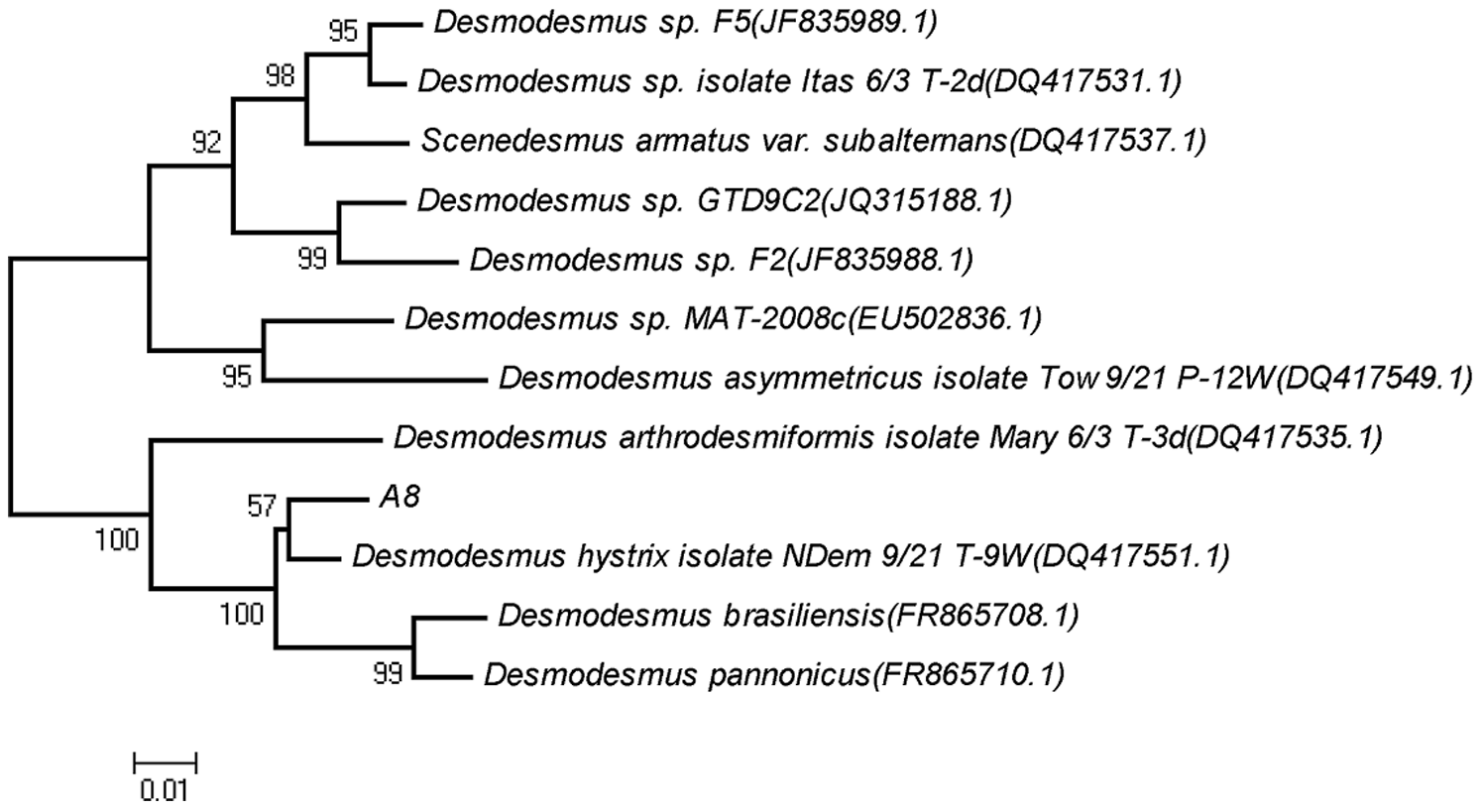
Phylogenetic tree of strain A8 and closely related species based on ITS sequences of ribosomal DNA. The tree was constructed using the neighbor-joining method. The numbers at nodes indicate the percentages of occurrence of the branching order in 1000 bootstrapped trees for values greater than 50%. Scale bar = 1% divergence.

The ITS1 and ITS2 sequences are important alternative markers for investigating the phylogenetic relationship within the *Scenedesmaceae* [26]. To assess the molecular diversity of the morphotype represented by strain A8, the 18S rRNA gene–ITS1–5.8S rRNA gene–ITS2 regions were sequenced. The length of the sequence amplied was 714 bp, including 27 bp 18S rRNA gene, 213 bp ITS1, 181 bp 5.8S rRNA gene, 280 bp ITS2, and 13 bp 28S rRNA gene. The sequence in this study has been deposited in the GenBank database under accession number JQ973888. BLAST was applied to find regions of similarity between biological sequences. The results showed that strain A8 should indeed be assigned to the genus *Desmodesmus* and was most closely related to *Desmodesmus hystrix* isolate NDem 9/21 T-9W DQ417551 (98% sequence similarity), *Desmodesmus brasiliensis* FR865708 (96% sequence similarity), and *Desmodesmus pannonicus* FR865710 (94% sequence similarity). These three strains formed a distinct subcluster in the neighbor-joining, in which the new isolate and *Desmodesmus hystrix* isolate NDem 9/21 T-9W DQ417551 formed a distinct subline (Figure 5).

### Contribution of A8 to Current Generation

**Figure 6:**
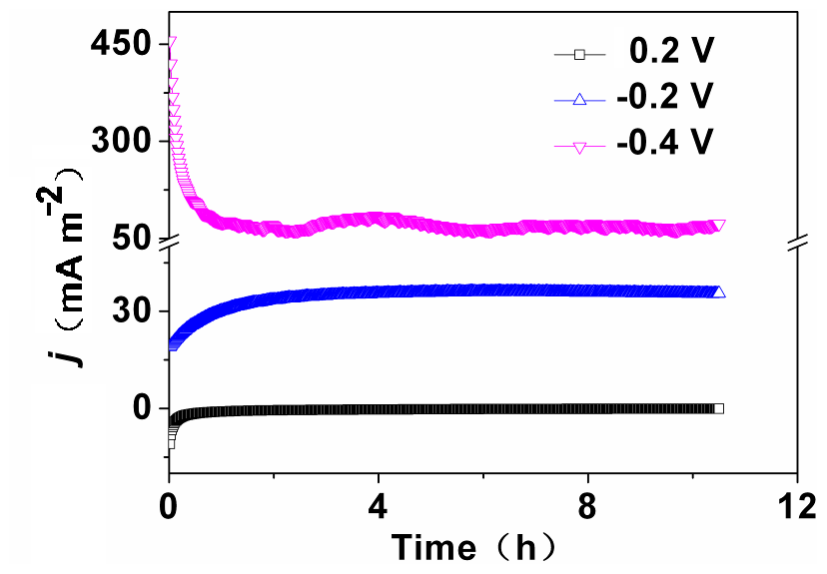
Current–time responses of strain A8 at potentials of +0.2,−0.2 and −0.4 V versus Ag/AgCl under illumination.

The function of A8 for current generation under illumination was evaluated by holding potentials of −0.4, −0.2, and +0.2 V vs Ag/AgCl. The setup was autoclaved and then filled with sterile BG11 and strain A8 (OD_680_ = 0.4) as inoculum. The current generation was clearly related to potential as shown in Figure 6. The current density at −0.4 V was 170 mA m^−2^ for *Desmodesmus* sp. A8, while the control setup generated a current density of 109 mA m^−2^ (Figure S4 in File S1). When the potential was increased to −0.2 V, the current density decreased to 35.9 mA m^−2^, which further decreased −0.24 mA m^−2^ as the potential was shifted to +0.2 V (Figure 6). Wang et al reported the effect of potential on current generation for an aerobic biocathode [27]; a low potential can generate high current for oxygen reduction reaction.

### Effect of Illumination on Current Generation

**Figure 7:**
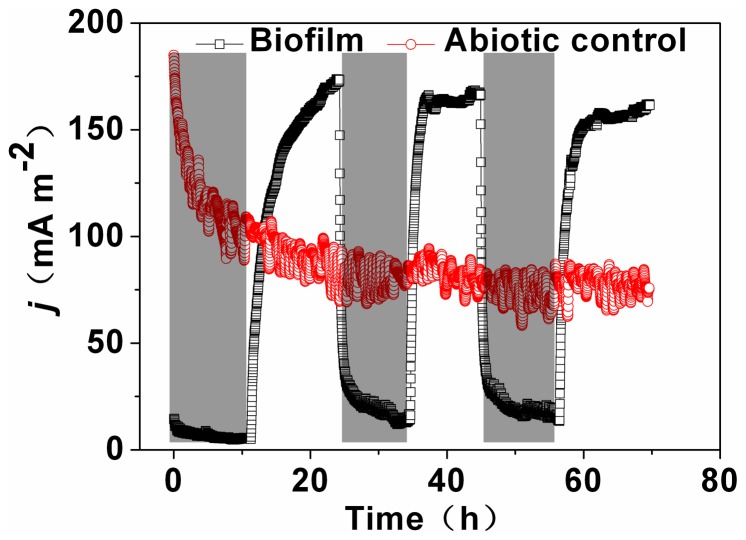
Current responses to light and dark at −0.4 V. The grey shaded zones indicate dark conditions.

Light supply is one of the most important factors that can affect the photosynthesis efficiency and metabolic pathways of microalgae [28]. Therefore, light should affect the performance of A8 incubated biocathode. In this study, current generation was compared in light and dark. The current density gradually increased from 17.7 to 164 mA m^−2^ under illumination in the first cycle. As the light turned off, the current rapidly decreased to 18.3 mA m^−2^. For the control, no current change was observed (Figure 7). As previously reported, DO concentration in the cathode is a key factor that can affect the performance of the cathode. Light supplies to the microalgae can accelerate the photosynthesis reaction, produce more oxygen for the cathode as electron acceptor, and thus enhance the current output. The same phenomenon was also observed by other research groups [28], [29].

**Figure 8:**
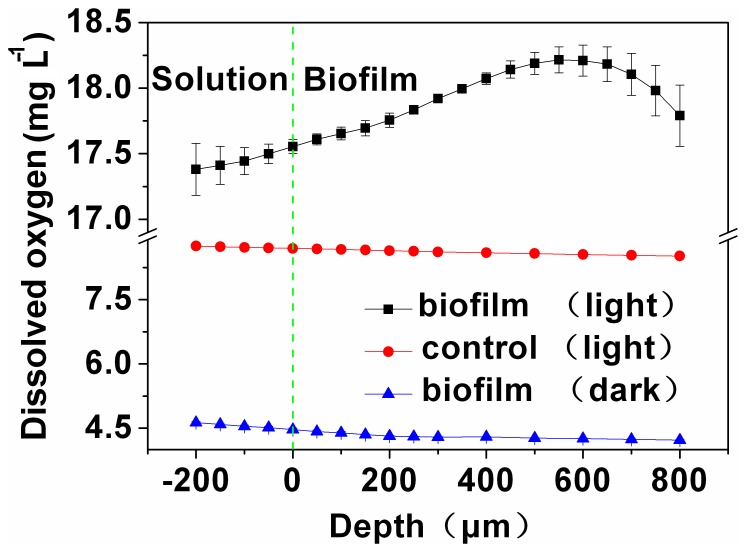
Profiles of dissolved oxygen within the A8 biofilm.

In the study, DO concentration was also determined using a microelectrode technique to get comprehensive understanding on how illumination affects the activity of algae at the micro-scale. Accompanying with the change in current, the DO rose to 18.3 mg L^−1^ under illumination, more than twice the saturated DO in deionized water (Figure 8), in agreement with Xiao et al. [30]. While in the dark, the DO concentration dropped to 4.22 mg L^−1^, lower than that of the control in light and suggesting that oxygen was consumed. Moreover, the DO concentration changed with the biofilm depth. In light, the DO apparently increased from 17.4 to 18.2 mg L^−1^ as the biofilm depth increased from −200 to 550 µm, and then decreased to 17.8 mg L^−1^ as the depth further increased to 800 µm. However, the DO within the biofilm decreased from 4.63 to 4.22 mg L^−1^ in the dark. For the control under illumination, the DO decreased from 8.75 to 8.52 mg L^−1^ as depth increased. These results demonstrated that A8 contributed to the DO concentration under illumination, which was dependent on the thickness of the biofilm. Under illumination, light energy was captured by chlorophyll antennae of algae, to split water with oxygen release [31]. Light intensity decreased as the biofilm depth increased, resulting in different amounts of energy captured by microalgae and thus different amounts of oxygen generated. The oxygen produced tended to diffuse out from the biofilm to the bulk solution. Therefore, the DO concentration first displayed an increasing gradient, but as the biofilm depth further increased, insufficient light for photosynthesis and oxygen consumption by microalgae/electrode resulted in the decreasing DO concentration.

DO is, in general, a limiting factor for biocathodes using oxygen as electron acceptor. One difference between microalgae and bacteria is the function of oxygen production. The DO gradient was positively related to the current; an anoxic zone near the electrode surface would ultimately be achieved for bacteria system [27], but would be overcome by microalgal biocathode systems.

## Conclusions

*Desmodesmus* sp. A8 displayed an oxidation peak in the potential range of +100 to +200 mV in cyclic voltammogram. The results confirmed that *Desmodesmus* sp. has the ability to transfer electrons to the electrode via electro-active proteins located on the cellular surface or via secreted oxygen. Illumination was shown to affect the current output due to the influence on oxygen generation by A8 cells. Hence, *Desmodesmus* sp. A8 is able to act as a cathodic microorganism under illumination.

## Supporting Information

File S1 **Supporting Figures.** Figure S1. Cyclic voltammograms of supernatant under anaerobic conditions. Figure S2. AFM image of *Desmodesmus sp.* A8 immobiled sodium alginate coating on glass slide. Figure S3. 3-dimension image of *Desmodesmus sp.* A8 immobiled sodium alginate coating on glass slide. Figure S4. Current –time (i-t) responses of *Desmodesmus sp.* A8 and control (A8 free carbon felt) under illumination at −0.4 V vs Ag/AgCl.
(DOC)
