# Supplementary material for: Isolation, Identification and Characterization of an Electrogenic Microalgae Strain
Source: PLoS One. 2013 Sep 3;8(9):e73442. doi: 10.1371/journal.pone.0073442 (PMC3760914; doi:10.1371/journal.pone.0073442)
Supplement: File S1 — Supporting Figures. Figure S1. Cyclic voltammograms of supernatant under anaerobic conditions. Figure S2. AFM image of Desmodesmus sp. A8 immobiled sodium alginate coating on glass slide. Figure S3. 3-dimension image of Desmodesmus sp. A8 immobiled sodium alginate coating on glass slide. Figure S4. Current –time (i-t) responses of Desmodesmus sp. A8 and control (A8 free carbon felt) under illumination at −0.4 V vs Ag/AgCl. (DOC) [file pone.0073442.s001.doc]

**Supporting Information**


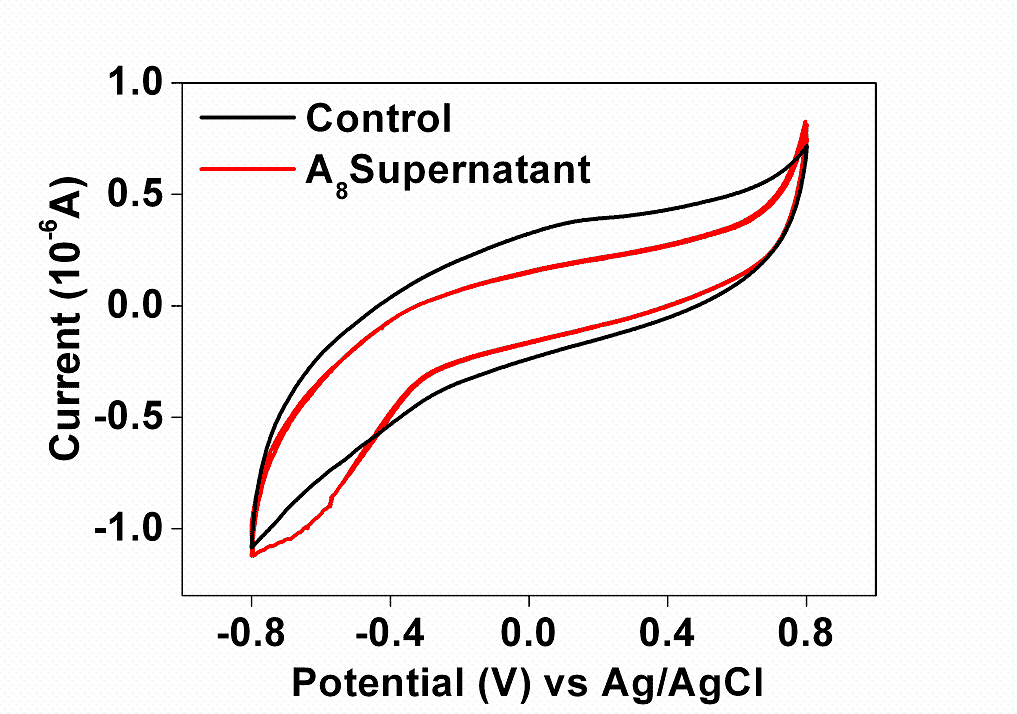


**Figure S1** **Cyclic voltammograms of supernatant under anaerobic conditions.**

**Topographic images**

The in situ atomic force microscopy measurement was performed using Ultra Objective Nanostation III (SIS GmbH). Topographic images were recorded in a non-contact mode. The time duration in obtaining one image composed of 256 lines was approximately 4.27 min at a scanning frequency of 1 Hz. The in situ optical microscope images were collected using a digital microscope (KEYENCE, VHX-600E), which attained a field depth of at least 20 times greater than that of traditional optical microscopes.

Strain A8 cell on the glass slide fixed by sodium alginate and CaCl_2_ was characterized by AFM to determine the size of the cells, as shown in Fig. S1 and S2. The height variation along a line in AFM images can be used to estimate the thickness of the sodium alginate and CaCl_2_ coating and strain A8 cell. The thickness of sodium alginate and CaCl_2_ coating was less than 800 nm, and the thickness of A8 cell in the coating was less than 700 nm. The Root-Mean–Square roughness parameter of A8 cell in the coating on an area of 100 μm^2^ was 142 nm.


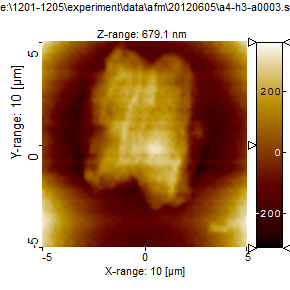


**Figure S2 AFM image of *Desmodesmus sp.* A8 immobiled sodium alginate coating on glass slide.**


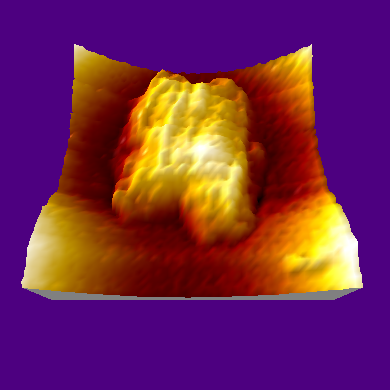


**Figure S3 3-dimension image of *Desmodesmus sp.* A8 immobiled sodium alginate coating on glass slide.**


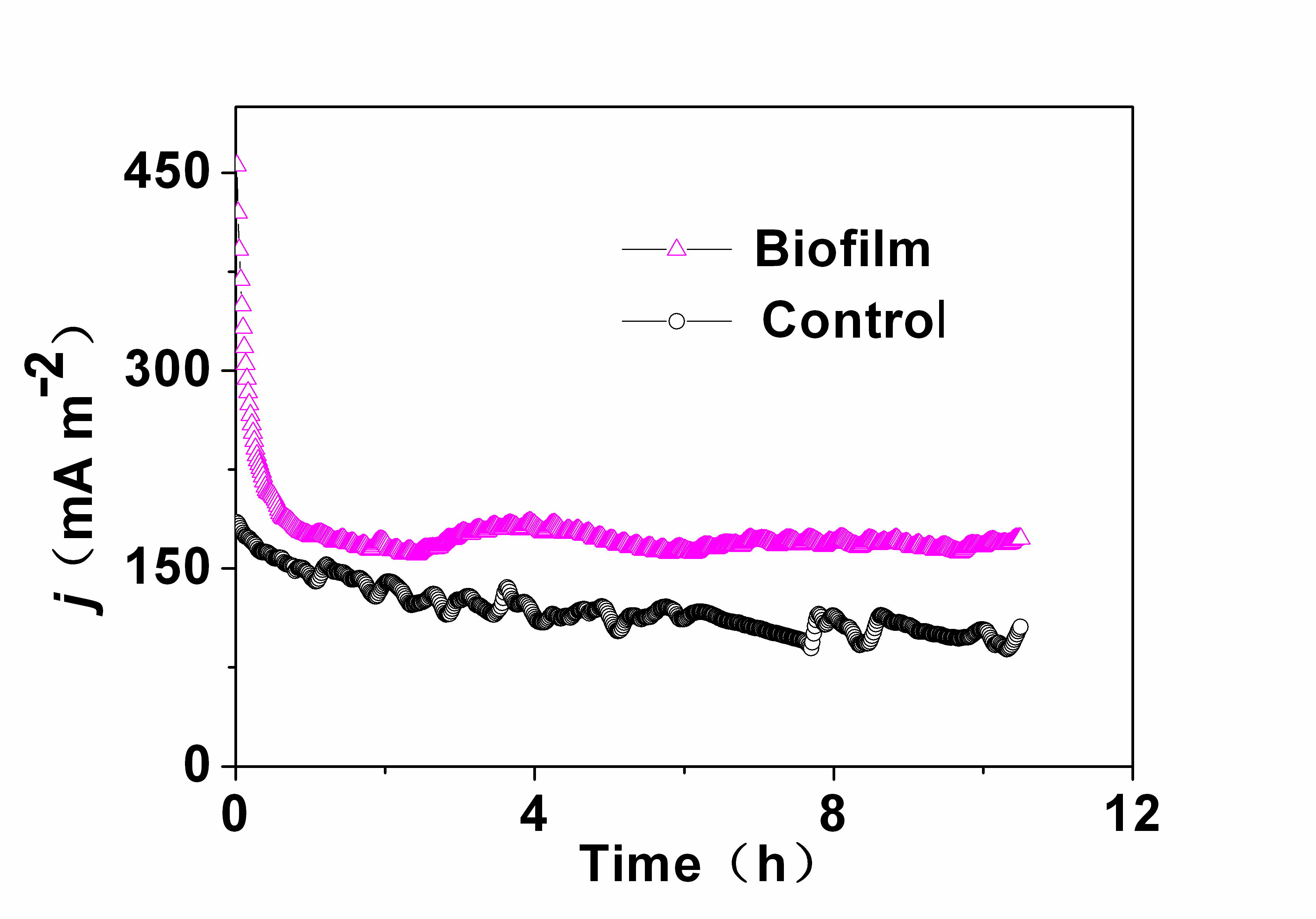


**Figure S4 Current –time (i-t) responses of *Desmodesmus sp.* A8 and control (A8 free carbon felt) under illumination at -0.4 V vs Ag/AgCl.**
